# Does a Low-Cost Act of Support Produce Slacktivism or Commitment? Prosocial and Impression-Management Motives as Moderators

**DOI:** 10.3389/fpsyg.2022.783995

**Published:** 2022-04-04

**Authors:** Lisa Selma Moussaoui, Jerome Blondé, Tiffanie Phung, Kim Marine Tschopp, Olivier Desrichard

**Affiliations:** ^1^Health Psychology Research Group, Faculty of Psychology and Education Sciences, Université de Genève, Geneva, Switzerland; ^2^Social Influence Research Group, Faculty of Psychology and Education Sciences, Université de Genève, Geneva, Switzerland; ^3^Faculty of Psychology and Education Sciences, Université de Genève, Geneva, Switzerland

**Keywords:** foot-in-the-door effect, blood donation, binding communication, sequential request, prosocial motivation, impression management (IM)

## Abstract

Increase or decrease in subsequent action following a low-cost act of support for a cause can be predicted from both commitment theory and the slacktivism effect. In this paper, we report on three studies that tested type of motivation (prosocial vs. impression management) as a moderator of the effect of an initial act of support [wearing a badge (S1) and writing a slogan (S2 and 3)] has on support for blood donation. Small-scale meta-analysis performed on data from the three studies shows that activating prosocial motivation generally leads to greater support for the cause after an initial act of support compared to the control condition, while the effect from impression-management motivation can either be negative or null.

## Introduction

Imagine that you are a manager of a blood donation center and you wish to launch a new communication campaign to make blood donation more visible and encourage people to donate more. In a meeting to prepare this campaign, you suggest distributing ribbons to people. Two psychology interns are attending the meeting; they each have a different opinion. The first one says that it is a great strategy because she learned from a lecture on commitment theory that, when people publicly display a low-cost support for a cause, the probability that they continue with consistent behavior (i.e., supporting the same cause) is higher. The other intern disagrees and says he recently read in an article addressing the slacktivism effect that, when people can show their support in public for a cause, it reduces the likelihood of engaging in subsequent behavior supporting that same cause. Who is right? Would you still take the risk of distributing ribbons, knowing that this may possibly have detrimental effects? In this paper, we report on three studies testing the potential of a moderating variable and the type of motivation (i.e., prosocial vs. impression management) to account for the increase or decrease in subsequent action following a low-cost act of support for a cause.

### Slacktivism: When an Initial Act Reduces Subsequent Action

Slacktivism is a portmanteau of “slacker” and “activism.” This term is used to describe “lazy activism,” or low-cost and low-risk support for a cause, which has almost zero impact on the cause itself (Morozov, 2009). Slacktivism can be expressed through social media (e.g., liking a post), although in this paper we also use the term slacktivism for real-life display of support, such as wearing a badge. Kristofferson et al. (2014) define slacktivism as token support that is marginally helpful toward for a cause, in contrast with meaningful support (e.g., tangible contributions such as giving money or time). One of the core implications of slacktivism, also known as the slacktivism effect, is that such low-cost display of support for a cause does not lead people to actively become involved in it and can even reduce subsequent commitment to acting for the same cause. The advertisement from the 2013 UNICEF campaign “Likes don’t save lives” shows that charities are aware of this problem: “Like us on Facebook, and we will vaccinate zero children against polio. We have nothing against likes, but vaccine costs money. Please buy polio vaccine at unicef.se. It will only cost you 4€, but will save the lives of 12 children.”

The slacktivism effect has been explained by a unique desire to fulfill a need in self-esteem (Lee and Hsieh, 2013; Kristofferson et al., 2014). By engaging in a low-cost action, especially in front of others, individuals show endorsement for a good and charitable cause. This places them in a favorable light, which can in itself boost their self-worth. In turn, since self-enhancement needs have been adequately satisfied, efforts for subsequent, more costly behavior appear useless to them. Studies on symbolic self-completion have shown that participants prevented from completing a task describing them positively tried harder to give a good impression to others (Wicklund and Gollwitzer, 1981; Gollwitzer et al., 1982). Thus, participants who are not given the opportunity to give a good impression to others can be predicted to try harder subsequently, compared to participants who had the opportunity to display that they are “a good person” by having performed a low-cost act of support.

Despite the popularity of the slacktivism phenomenon narrative (Morozov, 2009; Robertson, 2014), there is little empirical evidence of actual decrease in behavioral engagement after an initial low-cost act, as highlighted by Jones (2015). Strong evidence for the slacktivism effect would be the observation that participants who had publicly displayed their support for a cause engaged in less subsequent behavioral engagement than participants in a control condition of no initial act of support. Among the few empirical studies examining this question, Kristofferson et al. (2014) have interpreted the difference between private and public conditions as due to a slacktivism effect, but they found no significant difference between the initial act of support condition and the control condition (no initial act of support) in two of their studies (Study 1 and 3). One of the rare research studies showing reduction in activism after an initial act of support can be found in Schumann and Klein (2015). Compared to participants in the control condition (exposed to a website and then re-directed to the post-questionnaire), participants in the experimental condition (asked to post a comment on the website) were less willing to join an offline action.

Interestingly, a number of experiments studying slacktivism showed evidence of an increase in subsequent behavioral engagement after an act of support, especially when the subsequent action is related to the first cause (Lee and Hsieh, 2013; Kwak et al., 2018; Lane and Dal Cin, 2018). In fact, the prediction that an initial act of support should reduce the likelihood of a subsequent act is at odds with predictions raised by the commitment theory, which we will delve into in the next section.

### Commitment: When an Initial Act Increases Subsequent Action

According to the commitment theory (Kiesler, 1971; Joule and Beauvois, 1989), the fact that someone freely performs an initial action, in our case wearing a ribbon in support of blood donations, should increase the probability that he or she engages in a second related behavior such as volunteering for an event promoting blood donation. Studies provide support for the commitment-theory effect in various domains (Dillard et al., 1984), including blood donation (Hayes et al., 1984; Fonte et al., 2017), although this has not been consistently replicated in all studies [e.g., Gamian-Wilk and Dolinski, 2019; see Foss and Dempsey (1979) for an example in the field of blood donation].

Close to the subject at hand in this paper is the foot-in-the-door effect (Freedman and Fraser, 1966). This phenomenon, predicted by the commitment theory, occurs when making someone accept to engage in an initial low-cost behavior (called initial act of support) increases the chances of acceptance of a second, more costly behavior (Joule et al., 2007). The circumstances in which the behavior is engaged in are decisive for the individual to feel committed, notably that the person feels they are free to accept or refuse, and has no external justification for engaging in the behavior other than goodwill (Joule et al., 2007), costs of requests (Reingen and Kernan, 1977), the pro-social character of the request (Dillard and Hale, 1992) and delay between the two requests (Chartrand et al., 1999). In contrast to the slacktivism effect, commitment theory and the foot-in-the-door effect predict that a low-cost supportive action (a like on a post) should increase the likelihood that the person will adopt other behaviors to support the same cause. According to Chartrand et al. (1999), “the effect appears more often than would be expected by chance. Hence, the foot-in-the-door phenomenon first demonstrated by Freedman and Fraser (1966) is real. Unfortunately, this is only part of the story. […] The literature is filled with failures to replicate and occasional reversals of the effect” (p. 212). The same observation was made by Burger (1999), who noted that a large number of studies either failed to show increase, or found a decrease in compliance after a foot-in-the-door request compared to a control condition.

### A Moderator: Impression Management Vs. Prosocial Motivations

Research describes two antagonist effects, namely an increase or a decrease in behavioral engagement following an initial support action. These opposite predictions, opposing commitment theory to the slacktivism effect, have already been highlighted in previous studies (Lee and Hsieh, 2013; Kristofferson et al., 2014; Schumann and Klein, 2015; Lane and Dal Cin, 2018). One possibility for these predictions of opposite effects is that both slacktivism and commitment effects appear concomitantly. This can lead to neutralizing the impact of the low-cost action. But in certain circumstances it is possible that one of the two processes becomes more powerful than the other and causes an observable effect (positive vs. negative) of the low-cost action on subsequent behavior. In this paper, we propose to test the idea that when people are motivated to truly care for the cause, the commitment effect should occur more than the slacktivism effect, while, on the other hand, when they accept to carry out the initial act with the motivation of giving a good impression, then the slacktivism effect (i.e., a decrease in subsequent behavioral engagement) would arise more than commitment. We refer to impression-management motives in the latter case, and prosocial motivation for the former.

Impression-management (IM) motives, as discussed by Leary and Kowalski (1990), correspond to the desire to control one’s personal image and to show oneself to other people in a desirable way. This motivation has been mostly studied in the context of organizational citizenship behavior such as helping co-workers (Bolino, 1999). Interestingly, Hui et al. (2000) showed that employees who considered that organizational citizenship behavior would help them reach a goal (a promotion) made more such behavioral engagement, but also cut back on them after the goal was attained. Thus, a seemingly helping behavior can be enacted with a motivation other than helping.

The other side of the coin is prosocial motivation (PSM), which signifies that the reason behind engagement in a behavior is to benefit someone (Grant and Berg, 2010). The closeness between PSMs and altruism, as well as other concepts, have been discussed elsewhere (Schott et al., 2019). Given the extended debate on those terms (Cialdini et al., 1997; Batson, 2017), including in the field of blood donation (Evans and Ferguson, 2014), we take here the position of sticking to the term “prosocial.” According to literature in organizational psychology, PSM is opposed to IM as an explanation underlying the engagement in a given behavior, in one case to “do good” and in the other case to “look good” (Grant and Mayer, 2009).

Two papers have examined the role played by IM motives on the slacktivism effect. First, Lane and Dal Cin (2018) measured a score of IM before and after having asked participants to share a video promoting a social cause on Facebook, in two experimental conditions (i.e., public sharing vs. anonymous sharing). Contrary to their hypothesis based on slacktivism, they found that publicly sharing a video on social media increased willingness to volunteer. They also found that IM was not a mediator of the effect of publicly vs. anonymously sharing the video on willingness to support the cause. Second, Kristofferson et al. (2014) argued that a private condition (i.e., participants receiving a pin in a small envelope) should activate consistency motivation, while a public condition (i.e., participants being requested to wear a pin conspicuously on their coat or shirt) should activate IM motivation. The idea is that participants who would have already displayed their support for the cause in public would no longer need to engage in subsequent behavior (i.e., donate money). This reasoning is not consistent with commitment theory, which predicts that if an initial act is made in public, people are more committed to act in line with it and to engage further in subsequent related behavior. Thus, the public vs. private experimental manipulation does not make it possible to determine which of the competing predictions is accurate, i.e., whether there is increase or decrease in subsequent behavioral engagement after an initial act of support. Moreover, the Kristofferson et al. (2014) study did not test the effect of PSM, which we address in the present research.

### Our Research

Our research aims to explain why an initial low-cost, low-impact act of support for a cause leads to either more or less behavioral engagement. Our hypothesis is that the type of motivation by which the person engages in an initial act of support acts as a moderator. We predict that, if an individual is motivated to help a cause for prosocial reasons when agreeing to wear a ribbon or sign a petition (i.e., PSM), he or she would be more committed to contribute to the same cause when later asked to do so (H1). On the other hand, if acceptance of the initial act is driven mainly by desire to make a good impression (i.e., IM), then signaling that one is in favor of a cause *via* an action that is visible but that has no real impact could be enough to fulfill this motive, and the person will be less inclined to take other actions in support of the cause (H2). Consequently, we predict that the effect predicted by commitment theory (i.e., increased behavioral engagement after an initial act compared to control conditions) is driven by PSM, while the slacktivism effect (reduced behavioral engagement after an initial act vs. control) is driven by IM.

To test this hypothesis, we experimentally manipulated IM and PSM. In a two-by-two between-subjects design, participants were randomly assigned to a PSM condition or an IM condition. They were also randomly assigned either to an “initial act of support” condition or to a control condition (i.e., with no initial act of support). We then measured behavior (Study 1 and 3) and/or behavioral intention (Studies 2 and 3). Three studies were conducted following this experimental design, all about blood donation. We then summed up the results of the studies in a small-scale meta-analysis. We report how we determined our sample size, all data exclusions, all manipulations, and all measures for all studies (Simmons et al., 2012). The research project was approved by the University ethics committee (n° PSE.20190108.MM).

## Study 1

We sought to conduct a first test of our hypotheses in a field experiment on the university campus. There were three stages in this study. The first was to induce PS and IM motivations through pre-tested vignettes that participants had to imagine themselves into. The second stage was to have the participant engage in an act of support for blood donation, while the third stage measured intention to perform behavior related to blood donation. As a manipulation of initial act of support, we asked them to wear publicly a badge advocating blood donation. They were then invited to engage in a public awareness campaign for promoting blood donation among students. Our hypotheses were as follows:

H1: When the PS motivation was induced, we expected participants who received a badge to engage more in the campaign (vs. control).
H2: In contrast, when the IM motivation was induced, we expected participants who received a badge to report less engagement (vs. control).

### Methods

#### Participants

Given, based on the scarce literature on the subject, that estimating the expected effect size (ES) was difficult, we decided to follow the heuristic of Simmons et al. (2013), using a minimum 50 participants by condition, i.e., 200 in total. Of these, we excluded participants whose age was below 18 (*n* = 4), those who refused consent (*n* = 5), those who refused the badge (*n* = 1) and those who did not fully complete the study (*n* = 6). Our final sample thus included 184 participants.

The average age was 22.79 (*SD* = 5.11), ranging from 18 to 53 years old. There were 131 women and 53 men. They were randomly allocated to one of the four conditions of a 2 (initial act of support: yes vs. no) × 2 (PSM vs. IM motivation) between-subject factorial design.

#### Procedure and Materials

Participants were recruited on the campus of a Swiss university where communication campaigns are regularly conducted to promote blood donation among students. To avoid any influence from peers when completing the study, participants were approached only when seated at a table by themselves. The research assistant^1^, specifically trained to run this study, introduced herself as a master’s student looking for people to participate in one short study involving reading a text and answering a couple of questions. Participants were informed that participating in the study would enter them into a prize draw with the chance of winning a voucher worth 50 Swiss francs. Once they agreed to participate, the assistant provided them with either the PS or IM vignette at random, with instructions to read it and then list three thoughts they might have had in mind while reading (the full vignettes are provided in Electronic Supplementary Material 1).

**FIGURE 1:**
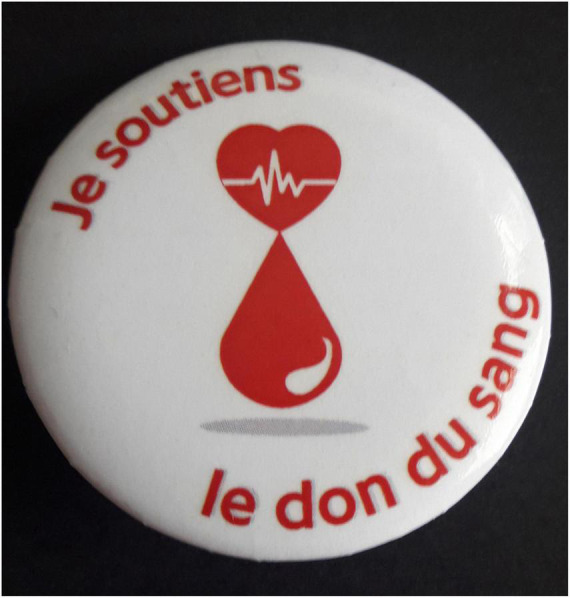
Badge used in the initial act of support condition in Study 1. The text is in French and means “I support blood donation.” Designed by the studio Atelier du Badge, using a logo taken from freepik.com. The badge size is 37 mm.

After thanking participants for completing the study, the research assistant informed them that she was concomitantly working as part of an internship for a Swiss blood donation organization and that one aspect of her work was to sensitize students about the importance of donating blood. In the initial act of support condition, participants were asked if they would agree to help the organization support blood donation by wearing a badge showing explicitly that they are supportive for this cause. What constituted our binding act was accepting this request. The badge was relatively small in size, white, and mentioned in red “I support blood donation” (see Figure 1). They were told that they could pin it on them wherever they wanted to.

In accordance with the principles of the commitment theory (i.e., stating that commitment effects occur only if a feeling of freedom may be guaranteed), they were specified that they were free to comply or to decline wearing it if they did not want to Joule and Beauvois (1998). This procedure has been previously used in a great deal of studies (e.g., Kilbourne and Kilbourne, 1984; Girandola et al., 2007). Only one participant refused the badge. Afterward, the research assistant informed the participant that a (fictitious) promotion campaign aimed at increasing students’ awareness of the benefits of blood donation would be taking place soon on the university campus and would be administered by the organization she works with. As a measure of subsequent behavior, we asked them if they would accept to lend a hand for a few hours in implementing this campaign (which involved talking with students about why blood donation is important); this was done by providing them a registration form indicating “I want to volunteer for the campaign: YES/NO” and then asking for their name, email address and signature. We considered that participants were willing to participate only if they entirely filled in and signed the form.

In the control condition, participants were given the same information as in the experimental condition but were not offered to wear any badge. The research assistant only proposed them to fill in the registration form.

One week later, participants were sent an email which requested them to answer questions about the duration they wore the badge and to give consent to use their data. We also debriefed and thanked them for their participation.

#### Pilot Study

The vignettes were pre-tested in a pilot study and compared with two other types of manipulations (i.e., first-person essays, and third-person essays). With one PS motivation condition and one IM motivation condition for each manipulation, we made comparisons between six experimental conditions in total and planned on choosing the manipulation with the highest between-condition differences in self-reported PS and IM motivations. Participants in this pilot study (*N* = 121; including 66 women) were randomly assigned to one of the six conditions before being asked to answer measures of PS and IM motivations (which are both described in Study 2 as manipulation checks). In the vignettes, we asked participants to read a hypothetical scenario by trying as much as possible to imagine themselves as if they would be experiencing the situation described and put personally in the main character’ shoes. The IM motivation vignette described a job interview where someone was depicted as putting efforts into making a good impression to the interviewer, while the PS motivation vignette described a situation where someone is giving assistance to an old person (for a previous use of vignettes, see e.g., Grant and Berry, 2011, Study 3). In the essays, participants were instructed to write about a situation or personal event where they had been trying either (1) to help someone selflessly (i.e., the PS motivation condition) or (2) to look good to others (i.e., the IM motivation condition). Some had to write a first-person essay (i.e., imagine a situation that participants had personally experienced), others had to write a third-person one (i.e., imagine a situation that someone else could have experienced). We performed a series of *t*-tests comparing each PS condition with the corresponding IM condition on both PS and IM measures. Despite differences are only approaching significance, we found that the vignettes showed the strongest differences in means for both motivations. The PS vignette induced more PS motivation (*M* = 5.05, *SD* = 0.67) than the IM vignette [*M* = 4.68, *SD* = 0.78; Mean Difference (*MD*) = 0.36, *SE* = 0.22; *t(*42) = 1.67, *p* = 0.10], while the IM vignette induced more IM motivation (*M* = 3.54, *SD* = 1.02) than the PS vignette [*M* = 3.05, *SD* = 1.25; *MD* = −0.49, *SE* = 0.34; *t(*42) = −1.42, *p* = 0.16]. Consequently, we decided to retain vignettes as a manipulation for PS and IM motivations.

### Results

**FIGURE 2:**
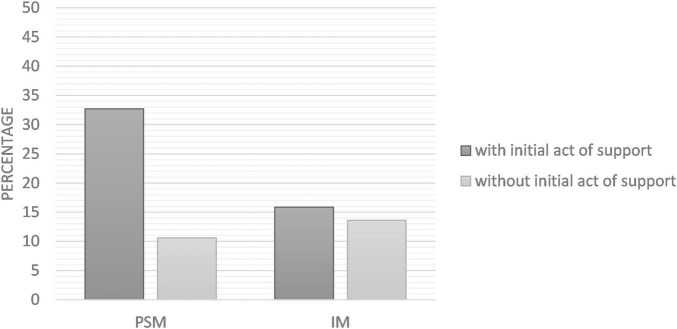
Percentages of volunteering acceptance according to experimental conditions (Study 1).

Logistic regression revealed that the interaction between motivation type and initial act of support (presence/absence) on behavior was not significant [Wald = 2.20, *p* = 0.138, *OR* = 3.40, 95% CI (0.67, 17.10)]. Two additional logistic regressions were run to test the effect of the initial act of support in each condition of motivation. Data supported our first hypothesis (H1): in the PSM condition, we found that the initial act of support had a significant positive effect on volunteering, Wald = 6.23, *p* = 0.013, *OR* = 4.07, 95% CI [1.35, 12.27]. In the IM condition however, the initial act of support did not have any effect on volunteering, Wald = 0.09, *p* = 0.764, *OR* = 1.20, 95% CI [0.37, 3.90]. Thus, the hypothesis of a decrease in subsequent behavioral engagement after an initial act of support in the IM condition (H2) was not supported. Percentages of acceptance to volunteer according to experimental conditions are presented in Figure 2.

Analyses of the follow-up data revealed that 68% of participants who accepted the badge did not actually wear it. Ten percent mentioned not having received one (despite being in the badge condition). To those who said they had worn the badge, we asked for how long they did so. The lowest duration was 5 h/the whole day (four persons), and nearly all the others reported still wearing it 1 week later.

### Discussion

In Study 1, we found that, when PSM was induced, an initial act of support (accepting a blood donation badge) produced an increase in acceptance of the subsequent behavior (spending time at a blood donation promotion event) in comparison with a control condition. This result is consistent with the idea that the foot-in-the-door effect occurs particularly when there is a strong PSM. However, as the effect of the initial act of support was non-significant in the IM condition, this precludes giving support to the slacktivism effect. The low level of volunteering in both conditions of IM might have induced a floor effect, preventing the appearance of a slacktivism effect. Most participants did not wear the badge, according to their answers in the follow-up survey 1 week later. This result is not an issue in itself, as the dependent variable (DV) was measured just after badge acceptance, but it suggests that badge distribution is not the optimal low-cost act of support.

## Study 2

In view of the results of Study 1, we decided to conduct a second study to test our hypotheses in a different experimental setting. Several substantial changes in the current study were thus made. First, since many participants may not have worn the badge, we changed the initial act of support in a way to ensure that it was performed by those who had accepted. Adopting a method frequently used in past research (Girandola and Joule, 2012; Demarque et al., 2013), we asked participants to make proposals for slogans in favor of blood donation. Second, rather than using an indirect measure of blood donation behavior, we directly measured intention to donate blood. Because the first study did not delve into the underlying mechanisms, we additionally included a measure of information processing with the idea that the effects of an initial act of support in the PSM condition (H1) would be driven by thorough processing of why blood donation is an important cause, while the effects of an initial act of support in the IM condition (H2) would be underpinned by shallower processing, notably because people are only motivated to make a good impression to others and do not really pay attention to blood donation in itself. Third, we controlled for past donation behavior, as this has been shown to affect intention to donate blood (Ferguson and Bibby, 2002; Godin et al., 2005), and we excluded those participants who cannot donate for medical reasons.

### Methods

#### Participants

The interaction ES obtained in Study 1 was used in G-power (Faul et al., 2007) to estimate the required sample size for Study 2^2^, resulting in a required sample of 117. However, given the variation in material and settings for the second study, and in anticipation of an unknown number of exclusions, we chose to stick to the rule of thumb of 50 participants in each condition as used in Study 1, and thus planned to recruit 200 participants for this study. Due to the fact that the platform used for recruitment allows new participants to begin a survey before others have finished, 285 participants started the study, but only 199 answered all questions. We excluded people who were unable to donate blood (*n* = 31), those who refused consent (*n* = 2), and those who did not read the vignette long enough (i.e., 24 s was estimated as a minimum to read it correctly; *n* = 21). This left a final sample of 145 participants. The average age was 30.70 (*SD* = 9.88), ranging from 18 to 71 years old. There were 70 women and 75 men. Sixty-two participants had already donated blood at least once (42.8%). Participants were randomly assigned to one of the four conditions of a 2 (initial act of support: yes vs. no) × 2 (motivation: prosocial vs. impression management) between-subject factorial design.

#### Procedure and Materials

Participants were recruited on Prolific in exchange for money (£0.90 for a 10-min survey). After introducing the study as being about blood donation, participants were randomly assigned to read one of the two vignettes (same as in Study 1) and then had to list the thoughts they might have had while reading it. They were, however, told that this part of the study was unrelated to the rest and consisted of a short but necessary methodological break.

In the initial act of support condition, participants were informed that we were about to implement a large communication campaign about blood donation with the support of the organization we allegedly worked with. They were told that we needed their help in finding catchy slogans that might convince people to donate blood and that we would use the slogans for designing the upcoming national campaign. To strengthen this scenario, we wished to make the initial act of support public by specifying that all the proposals would be available on the organization website and that people could vote for the best slogan to be used in the campaign. Accordingly, participants were then instructed to write one short and persuasive slogan, as well as one personal and unique handle that was supposed to appear right next to their slogan. Afterward, we measured intention to donate blood and information processing. In the control condition, participants were not requested to find a slogan. After the motivation manipulation, they only had to complete the measures.

Intention was assessed with three items, e.g., “I intend to donate my blood in the next 4 months” (α = 0.96). Responses were given on 7-point rating scales, ranging from “Not agree at all” to “Totally agree.” Information processing was measured by using a thought-listing task. Participants were asked to write down all the thoughts they had about blood donation, such as benefits and motives for donating blood. They were requested to report 1–5 thoughts in total. Depth of information processing was calculated by summing all the thoughts participants had reported (*M* = 3.08, *SD* = 1.40). After this, we included manipulation checks using five items from the prosocial values and impression management subscales of the Citizenship Motives Scale (Rioux and Penner, 2001; PS motivation: α = 0.87; IM motivation: α = 0.82), and then asked participants to provide socio-demographic information (i.e., gender, age, past donation behavior). Finally, they were briefly debriefed, thanked for their participation, and asked for their consent.

### Results

Manipulation checks were analyzed with 2 × 2 ANOVAs estimating main effects of motivation and act of support, as well as their interaction. This showed that participants in the PSM condition had higher scores on the prosocial items (*M* = 5.08, *SD* = 0.09) than participants in the IM condition (*M* = 4.80, *SD* = 0.10), *F*(1,139) = 4.16, *p* = 0.043, η^2^_*p*_ = 0.03. Main effect of the act of support and interaction effect were both non-significant respectively, [*F*(1,139) = 0.59, *p* = 0.808, η^2^_*p*_ < 0.01; *F*(1,139) = 0.03, *p* = 0.854, η^2^_*p*_ ≤ 0.01]. Even though means are descriptively consistent with expectations, the difference on the IM items was not significant between the conditions of motivation, *F*(1, 141) = 1.94, *p* = 0.166, η^2^_*p*_ = 0.01, (*M_*PSM*_* = 3.43, *SD* = 0.11, *M_*IM*_* = 3.17, *SD* = 0.12), neither was the main effect of the act of support *F*(1,141) = 1.837, *p* = 0.185, η^2^_*p*_ = 0.01, nor their interaction, *F*(1,141) = 1.837, *p* = 0.177, η^2^_*p*_ = 0.01.

**FIGURE 3:**
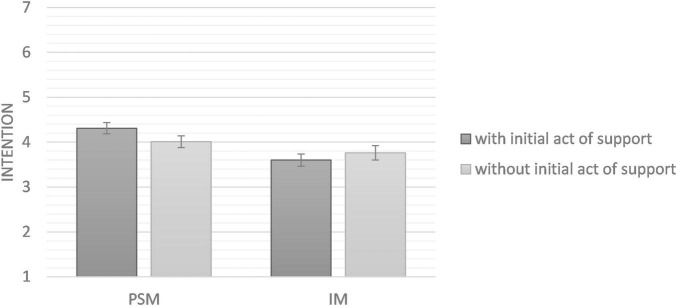
Means of intention to donate blood as a function of experimental conditions (Study 2). Error bars represent standard deviation.

An ANOVA showed that having donated in the past had a significant effect on intention to donate blood [*F* (1,140) = 60.03, *p* < 0.001, η^2^_*p*_ = 0.30, 90%^3^ CI (0.19, 0.38)]. However, the experimental manipulations did not interact with each other [*F* (1,140) = 0.72, *p* = 0.397, η^2^_*p*_ = 0.01, 90% CI (0, 0.04)]. Simple effects tested through ANOVAs revealed that neither H1 nor H2 were supported. In the PSM condition, intention did not significantly differ between the condition with the initial act of support and control condition, *F*(1, 80) = 0.60, *p* = 0.441, η^2^_*p*_ = 0.007, 90% CI [0, 0.07]. Similarly, in the IM condition, no significant effect emerged either, *F*(1, 59) = 0.16, *p* = 0.690, η^2^_*p*_ = 0.003, 90% CI [0, 0.06]. Results are presented in Figure 3.

Analysis of the number of thoughts listed related to blood donation showed that, in the IM condition, there was no effect from the initial act of support, *F*(1, 59) < 0.01, *p* = 0.997 (*M*_act of support_ = 2.95, *SD* = 0.25; *M*_control_ = 2.95, *SD* = 0.29). Similarly, in the PSM condition, the number of thoughts listed did not vary according to the experimental conditions, *F*(1, 80) = 0.05, *p* = 0.819, (*M*_act of support_ = 3.20, *SD* = 0.21; *M*_control_ = 3.13, *SD* = 0.21).

### Discussion

Study 2 did not replicate Study 1 in the PSM condition: the positive effect of the initial act of support was not found in Study 2. No effect of the initial act of support emerged when the IM motivation was activated, both in Study 1 and 2. Using behavioral intention as the sole DV in Study 2 might be a limitation, as DVs are mostly observed behavior in the literature on slacktivism and foot-in-the-door [e.g., Joule et al., 2004; Kristofferson et al., 2014; Studies 1 and 5; but see Fonte et al. (2017)]. It is possible that intention is an indicator of a more conscious level of processing and that our effects of interest are not captured by such DV. Another limitation is that the online setting might have rendered less credible the fact that the first and second part of the study were unrelated, and led participants to doubt our cover story. Finally, the power analysis based on Study 1 might not have been adequate, as the effect sizes issued from studies with small N can be part of a wide prediction interval and not replicate with the same magnitude in further studies (Patil et al., 2016). Thus, a more conservative effect size could have been used, and the absence of effect in Study 2 could also be linked to an insufficient statistical power.

## Study 3

Because we could not sufficiently back up our predictions thus far, we planned another study to address the limitations of the previous ones. The most notable changes were the general setting of the study and dependent variable. Similar to Study 1, this study was a field experiment conducted on the university campus by a research assistant^4^. However, unlike Study 1 but similar to Study 2, we asked participants to propose slogans promoting blood donation as a procedure to manipulate the initial act of support. This choice was made to prevent the issue of Study 1 related to participants not wearing the badge. We measured both intention and actual donation behaviors by recruiting participants 2 weeks before a real on-site blood donation drive took place on the university campus. One week after the drive, we asked them to report whether they had donated blood there or not. This study was preregistered on AsPredicted^5^. Our preregistration plans included a description of our hypotheses, dependent variables, possible adjustments, recruitment rules, exclusion criteria and our strategy for handling outliers.

### Methods

#### Participants

Similar to Study 1 and 2, an approximate target of 200 participants was aimed at. However, recruitment period was limited in time because of the forthcoming blood donation action, and only 190 participants were recruited. We excluded those who refused consent (*n* = 11), one whose age was below 18 (*n* = 1), participants who stated they were unable to donate blood (*n* = 18), and participants who did not complete the second part of the study (*n* = 6). The final sample was thus made up of 154 participants. The average age was 22.53 (*SD* = 5.14), with a range of 18–47 years old. There were 51 women and 101 men (2 reported incoherent gender on both parts of the study). There were 48 participants who reported having already donated blood at least once (31.2%). We randomly assigned participants to one of the four conditions of a 2 (initial act of support: yes vs. no) × 2 (motivation: prosocial vs. impression management) between-subject factorial design.

#### Procedure and Materials

We recruited participants on the university campus 2 weeks before an on-site campus blood donation action was proposed to students and university staff by the regional blood transfusion center. The procedure of this study was similar to Study 1 (including the same vignettes), except for the manipulation of the initial act of support, which was similar to Study 2 (i.e., participants had either to propose a catchy slogan or not). Right after this manipulation, we asked participants to answer whether they intended to donate blood in the next 4 months and whether they intended to donate blood during the on-site campus collection. Answers could be given on 7-point scales ranging from “Not at all” to “Yes, absolutely.” In addition, we measured gender, age, and past donations. One week later, a Qualtrics survey link was sent that further asked participants whether they actually did go donate blood during the on-site campus donation drive. Three answers were given: “Yes, I did” (coded = 1), “Yes, I tried but I was deferred” (coded = 1), “No, I did not” (coded = 0). We considered that donation behavior was actually performed as long as the participant attempted to donate, even if donation was denied for some reason by the transfusion center. At the end, they were debriefed, thanked for participating, and asked for consent.

### Results and Discussion

Zero-order correlations between each intention items and behavior were calculated. Intention to donate in the next 4 months correlated at 0.471 (*p* < 0.001) with donation behavior, and intention to donate the following week correlated at 0.385 (*p* < 0.001) with donation behavior.

Interactions and simple effects were computed in ANOVAs for both measures of intention and in logistic regression for the measure of behavior.

#### Intention to Donate in the Next 4 Months

**FIGURE 4:**
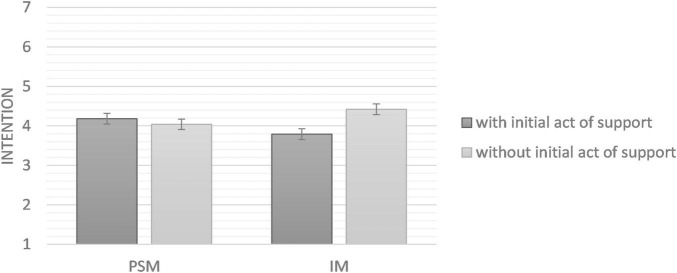
Scores of intention to donate blood in the next 4 months (Study 3). Error bars represent standard deviation.

The interaction on the DV intention of donating blood in the next 4 months was not significant, *F*(1, 148) = 2.02, *p* = 0.157, η^2^_*p*_ = 0.013, 90% CI [0, 0.06]. We did not find any effect of the initial act of support in the PSM condition (H1), *F*(1, 75) = 0.04, *p* = 0.837, η^2^_*p*_ = 0.001, 90% CI [0, 0.03], or in the IM condition (H2), *F*(1, 72) = 3.33, *p* = 0.072, η^2^_*p*_ = 0.044, 90% CI [0, 0.14]. Results are presented in Figure 4.

#### Intention to Donate the Following Week

**FIGURE 5:**
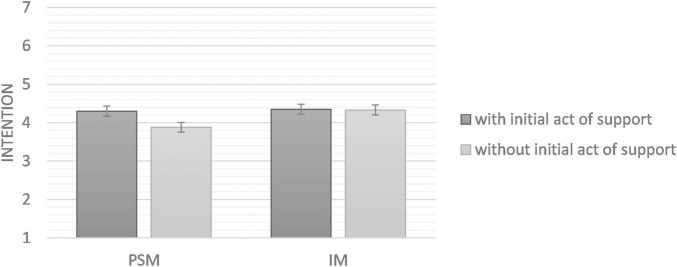
Scores of intention to donate blood next week (Study 3). Error bars represent standard deviation.

The interaction on the DV intention of donating blood the following week was not significant, *F*(1, 145) = 0.56, *p* = 0.457, η^2^_*p*_ = 0.004, 90% CI [0, 0.04]. Our results also did not show any effect of the initial act of support in the PSM condition (H1), *F*(1, 73) = 1.15, *p* = 0.286, η^2^_*p*_ = 0.016, 90% CI [0, 0.09], or in the IM condition (H2), *F*(1, 71) = 0.01, *p* = 0.938, η^2^_*p*_ = 0.000, 90% CI [0, 0.01]. Results are presented in Figure 5.

#### Behavior

**FIGURE 6:**
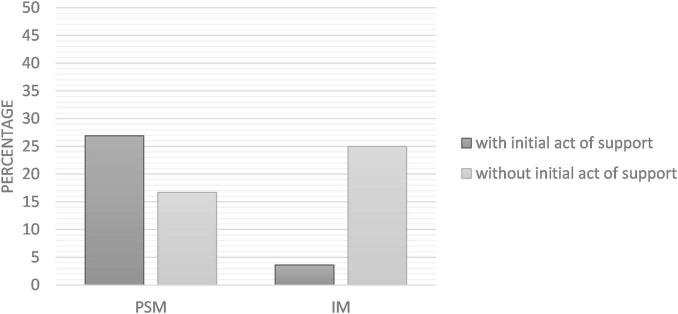
Percentages of participants who donated blood (self-report) according to experimental conditions (Study 3).

The interaction on the self-reported behavior was statistically significant, Wald = 4.48, *p* = 0.034, *OR* = 17.09, 95% IC [1.24, 236.43]. In the IM condition (H2), we found that participants who had written a slogan were significantly less likely to donate blood than those in the control condition [Wald = 4.10, *p* = 0.043, *OR* = 0.10, 95% IC (0.01, 0.93)]. In the PSM condition (H1), the initial act of support had no effect on donation behavior [Wald = 0.34, *p* = 0.559, *OR* = 1.53, 95% IC (0.37, 6.43)]. Results are presented in Figure 6.

Study 3 did not reveal significant effects of our experimental manipulation on the intention DVs. On the behavioral DV, in line with H2, participants in the IM condition exhibited less blood donation behavioral engagement when they had performed an initial act of support compared to those in the control condition. However, H1 was not supported.

## Small-Scale Meta-Analysis

Given inconsistencies in the results obtained across our studies, we decided to perform a small-scale meta-analysis (using random-effect models) to give a clearer picture of effects testing our hypotheses. We first converted *OR*s (Study 1 and 3) and means and standard deviations for the intention items (Studies 2 and 3) into Cohen’s *d* using the software Comprehensive Meta-Analysis (Borenstein et al., 2013). The two intention ES of Study 3 were averaged to enter only one weighted average ES into the meta-analysis (Maynard et al., 2007; Goh et al., 2016) using the formulas of Lipsey and Wilson (2001). We then used the software Rstudio (R Core Team, 2018) to run the small-scale meta-analysis, more specifically using the packages Metafor (Viechtbauer, 2010), Meta (Balduzzi et al., 2019), Esc (Lüdecke, 2018), and MAd (Del Re and Hoyt, 2010), this last package making it possible to take into consideration the multiple outcomes in Study 3 (both intention and behavior recorded). Aggregation of those outcomes taking into account their correlation (0.467) was performed using the syntax by Del Re (2015). We combined measures of intention and behavior in the same meta-analysis because intention was used as a proxy of behavior, and the validity of this link has been demonstrated for blood donation behavior notably (Schlumpf et al., 2007).

Subgroups analysis showed that in the PSM condition the effect of the initial act of support was in the positive direction, but small and with CIs including zero, *d* = 0.31, 95% CI [−0.01, 0.64]. In the condition with the IM motivation activated, the effect of initial act of support goes in the other direction, but is also of a small size and CIs includes zero, *d* = −0.26, 95% CI [−0.75 to 0.22]. The test for subgroup differences showed that the heterogeneity in the ES presented above is very close to the significance threshold, *Q* = 3.69, df = 1, *p* = 0.054. In order to complement those tests, we performed equivalence testing using the package Toster (Lakens, 2017). We used 0.2 as the smallest effect size of interest (based on Cohen rules of thumb). For both the effect sizes in PSM and IMP conditions, TOST concluded as non-significant, i.e., that we cannot reject H0 of the effect size being large enough to be deemed worthwhile. Interpretation would be that the effects are “undetermined (neither statistically different from zero nor statistically equivalent)” (Lakens, 2017, p.356).

**FIGURE 7:**
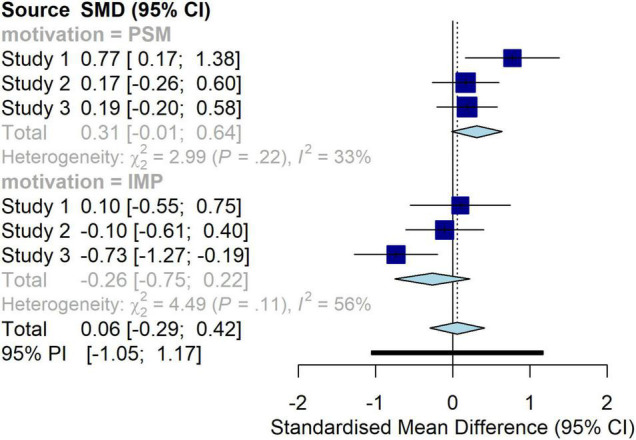
Forest plot of initial act of support according to the motivation condition.

Thus, the results of the small-scale meta-analysis are not unambiguous. Cautiously, there are indications that the initial act of support seems to have led to a stronger subsequent behavioral engagement among participants who were primed with a PS motivation, while the effect of the initial act of support for subsequent behavioral engagement among participants driven by IM motivations is either negative or null. Forest plots of the individual and overall ES are presented in Figure 7.

## General Discussion

Across three studies, we investigated the idea that motivation could explain why an initial act of support sometimes leads to investing more effort into the same cause (which is consistent with the commitment theory), and sometimes to investing less effort (which is consistent with the slacktivism effect). The small-scale meta-analysis enabled a better grasp of the results, although comparing results with equivalence testing led to conclude that the results are undetermined. There is a tendency suggesting that activating a prosocial motivation mostly engenders commitment for the cause after the initial act of support, while activating an IM motivation can sometimes lead to a reduction in investment in the cause after displaying support for it, but possibly also no effect, as the confidence intervals include zero.

Our results provide an interesting perspective helping to reconcile two lines of research that make two opposite predictions: the commitment theory on the one hand, and the slacktivism hypothesis on the other. The latter has received less empirical support than the former, and our results reflect that the ES of commitment is descriptively bigger than the ES of slacktivism. But occurrence of the latter does seem plausible and worth investigating, as its impact could be problematic at a time when much communication and activism happen online, and where it is extremely easy and frequent to display publicly and at no-cost one’s support for a cause.

Close to slacktivism are similar effects such as moral credits (Merritt et al., 2010), moral licensing (Khan and Dhar, 2006; Sachdeva et al., 2009) or negative spillover (Thøgersen and Ölander, 2003), all sharing the idea that, after performing “good” behavior, an individual might tend to relapse and perform badly. Those effects have attracted much attention, but empirical data is inconsistent. Across three high-powered studies, Blanken et al. (2014) sought to replicate the moral licensing effect. A meta-analysis including the original studies and the replications showed no significant effect. Other recent publications have reported failed replications (e.g., Moery and Calin-Jageman, 2016; Urban et al., 2019), and publication bias has been proposed as a possible explanation (Kuper and Bott, 2019). In our studies, the slacktivism effect was not consistently replicated; we thus believe that our data provide a contribution to this broader literature on relapse effects after a good behavior.

This paper can also be put in perspective with the study of Siem and Stürmer (2019). These authors showed that the intended publicity of a helping act influences the motives attributed to the helper: egoistic when the helping act is presented as purposefully public, and altruistic when presented as intended to be kept private. Did our participants have the meta-cognition of considering what others would think of them? Savary and Goldsmith (2020) showed that public recognition for donation can decrease donation through self-signaling: people do not want to appear as donors in order to get recognition. Future studies could explore how participants in commitment/slacktivism tasks think others see themselves, and how this impacts their behavior.

Additional mechanism could also be think of when considering the debate around the existence of “pure altruism” (Andreoni, 1990; Batson, 2017). Studies have shown that after a prosocial action the individual feel good about themselves, which categorize the act as benevolence instead of altruism (Ferguson et al., 2008). Using variants of the dictator game (an experimental task to measure choices) Ferguson et al. (2012) showed that charitable giving was driven by warm glow motivation. We can then suppose that, in our study setting, participants performing the initial act of support felt positive emotions because of their contribution. The subsequent effect of warm glow on persistence (i.e., similarly to H1) remain to be tested.

One of the strengths of our studies was to include measures of behavioral intention and actual behavior (both directly observed and self-reported) (Lewandowski and Strohmetz, 2009; Doliński, 2020), and the effects appeared more on the actual behavior DV than on measures of intention. Future studies could pursue the exploratory hypothesis that the level of awareness necessitating the answering of intention items might reduce the commitment and slacktivism effects.

We used two different types of initial act of support: wearing a badge (Study 1) and writing a slogan (Study 2 and 3). Those two acts vary on several dimensions: writing a slogan is irreversible, while the badge can be worn and then taken off. The public aspect is slightly different too, as the slogan was related to the person with a handle (thus only those who know who the handle designate can do the link), while for the badge, when it is worn, everyone can see it. Writing a slogan demand a cognitive effort, which may act as self-persuasion (Aronson, 1999; Miller and Wozniak, 2001), while accepting the badge involves only saying yes. Thus, although we consider both acts contain the element to trigger commitment and slacktivism, further research could investigate if specific dimensions are necessary for the initial act of support to have an impact.

In Studies 1 and 3, the same experimenter triggered the initial act of support and requested the subsequent behavior, which can be a limitation. In studies in the field of commitment theory, different experimenters have been used (Joule and Beauvois, 1998), and this was also the case in the studies conducted by Kristofferson et al. (2014). We acknowledge that it might be better to prevent participants from guessing the goal of the study, as it can lead to bias (but see McCambridge et al., 2012). In addition, this might provide the easiest conditions for detecting the slacktivism effect: as the initial act is performed in front of the experimenter A, IM motives related to this person are accomplished, while if experimenter B asks the request for subsequent behavior, the participant might want to re-establish a good impression on this new experimenter who would not have seen him or her performing the initial act of support. Thus, less slacktivism might occur if two different experimenters were to conduct the two parts of the studies, unless the initial act of support remains visible, such as by wearing a badge. This provides ideas for future studies. It is also a limitation that Study 1 and 3 were not double-blind, as the experimenter was aware of the experimental condition in which the participant was, as well as the hypothesis. To address this issue, future studies should be conducted with the aid of the research assistants unaware of the initial expectations, or by adding control condition in which we disclose the purpose of the study (vs. not) and test if the awareness of the study goal does impact the dependent variable.

The magnitude of effect size on the manipulation check (e.g., in Study 2) suggest the method induce only a small effect [in reference to Cohen’s rules of thumb (Cohen, 1992)]. Future studies could also test other ways to induce motivation, such as empathy activation *via* perspective-taking instructions (Stocks et al., 2009) for pro-social motivation or explicit instruction to make another person perceive the self in as positive a light as possible for impression-management motivation (Malle et al., 2007; DeAndrea and Walther, 2011; Study 6).

To conclude, we would like to tentatively formulate a recommendation for the blood donation center manager with whom we started this paper. In the light of our results, the nuanced answer is that the effect of distributing ribbons to people will depend on their own motivation toward blood donation. Not taking motivation into consideration might lead to an overall inefficient campaign, as slacktivism and commitment can cancel each other out.

## Data Availability Statement

Data are available on OSF at: https://osf.io/qeh6a/?view_only=6eca3885295043c49219cc41ccfd9c3d.

## Ethics Statement

The studies involving human participants were reviewed and approved by Commission Universitaire pour une Recherche Ethique à l’Université de Genève, from Geneva University (approval no PSE.20190108.MM). The patients/participants provided their written informed consent to participate in this study.

## Author Contributions

LM, JB, TP, and OD designed study 1. TP collected data for study 1. LM, JB, and TP analyzed data from study 1. LM, JB, and OD designed study 2. LM and JB collected and analyzed study 2 data, analyzed data from study 3, and wrote the manuscript. LM, JB, KT, and OD designed study 3. KT collected data for study 3. LM conducted the small-scale meta-analysis. TP, KT, and OD provided comments on the draft. All authors agreed on the final version of the manuscript.

## Conflict of Interest

The authors declare that the research was conducted in the absence of any commercial or financial relationships that could be construed as a potential conflict of interest.

## Publisher’s Note

All claims expressed in this article are solely those of the authors and do not necessarily represent those of their affiliated organizations, or those of the publisher, the editors and the reviewers. Any product that may be evaluated in this article, or claim that may be made by its manufacturer, is not guaranteed or endorsed by the publisher.

## Footnotes

1 It is important to note that this assistant was aware of the research hypotheses, thus the study is not double-blind.

2 *OR* = 3.4 was converted into partial η^2^ using https://www.psychometrica.de/effect_size.html. Partial η^2^ = 0.1022 was entered in the *a priori* analysis, ANCOVA menu, alpha = 0.05, power = 0.95, numerator df = 1, number of groups = 4, number of covariates = 1.

3 90% CI are used here because η^2^_*p*_ cannot have a negative sign; the test is thus one-sided (Steiger, 2004).

4 The research assistant was not blind to the experimental conditions and the research hypotheses.

5
https://aspredicted.org/blind.php?x=zq7r95
